# Supplementary material for: EGCG binds intrinsically disordered N-terminal domain of p53 and disrupts p53-MDM2 interaction
Source: Nat Commun. 2021 Feb 12;12:986. doi: 10.1038/s41467-021-21258-5 (PMC7881117; doi:10.1038/s41467-021-21258-5)
Supplement: Supplementary file 1 — Supporting Information [file 41467_2021_21258_MOESM1_ESM.pdf]

Supporting Information

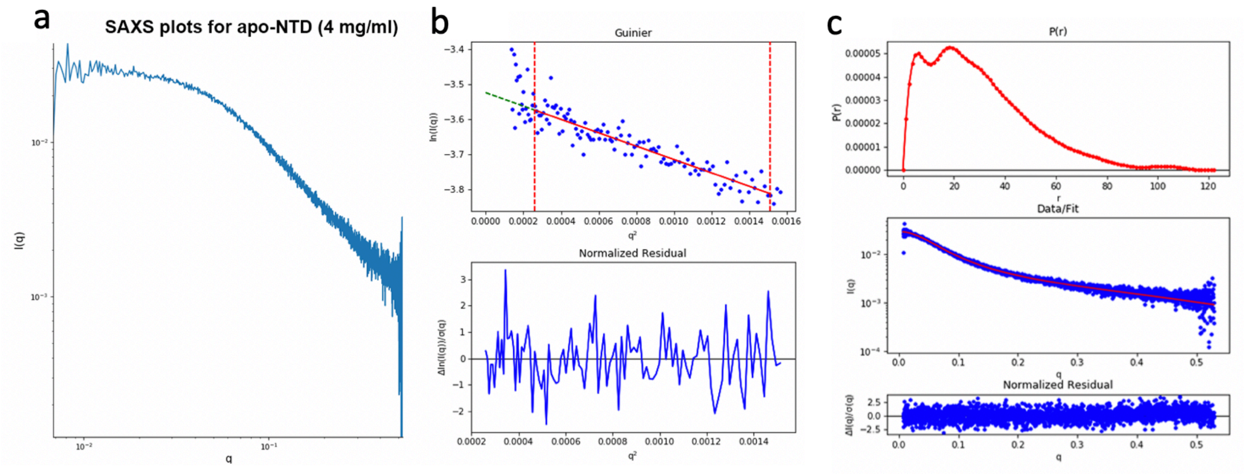

Figure S1. SAXS plots for apo-NTD. (a) The scattering intensity profile I (q) is shown as a function of the scattering vector (q). (b) The Guinier plot (top panel) is shown together with the normalized residual of the Guinier fit (bottom panel). (c) The pair distance distribution function p(r) is shown (top panel). The middle panel shows the scattering intensity profile calculated from the p(r) function (red) overlaid with the scattering intensity profile of the actual data (blue). The bottom panel shows the normalized residual of the fit.

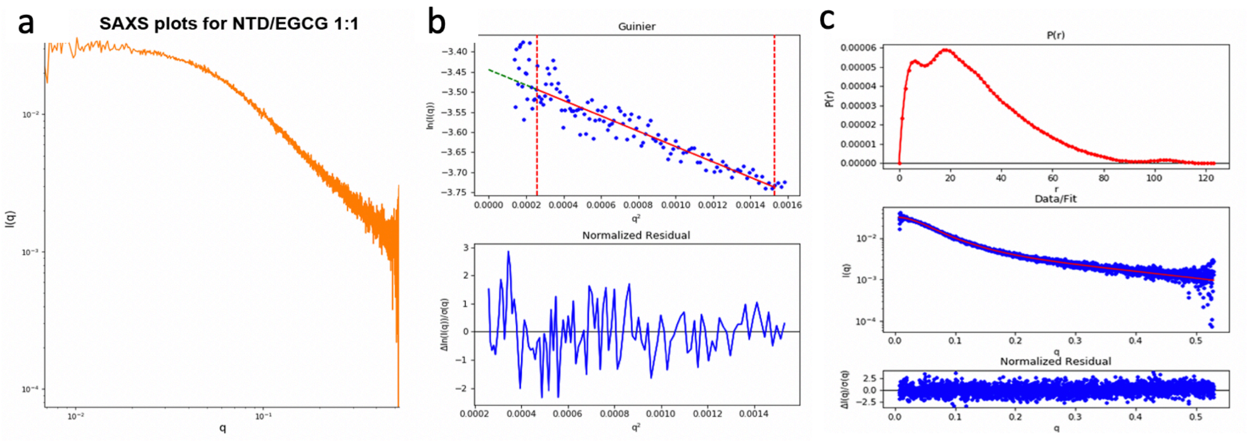

Figure S2: SAXS plots for NTD/EGCG mixed in 1:1 molar ratio. (a) The scattering intensity profile I (q) is shown as a function of the scattering vector (q). (b) The Guinier plot (top panel) is shown together with the normalized residual of the Guinier fit (bottom panel). (c) The pair distance distribution function p(r) is shown (top panel). The middle panel shows the scattering intensity profile calculated from the p(r) function (red) overlaid with the scattering intensity profile of the actual data (blue). The bottom panel shows the normalized residual of the fit.

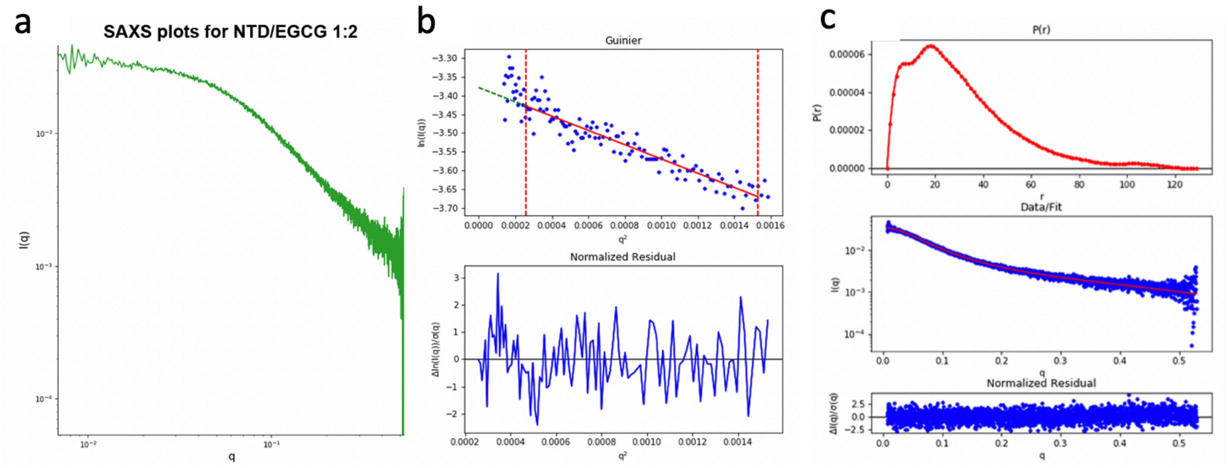

Figure S3. SAXS plots for NTD/EGCG mixed in 1:2 molar ratio. (a) The scattering intensity profile I (q) is shown as a function of the scattering vector (q). (b) The Guinier plot (top panel) is shown together with the normalized residual of the Guinier fit (bottom panel). (c) The pair distance distribution function p(r) is shown (top panel). The middle panel shows the scattering intensity profile calculated from the p(r) function (red) overlaid with the scattering intensity profile of the actual data (blue). The bottom panel shows the normalized residual of the fit.

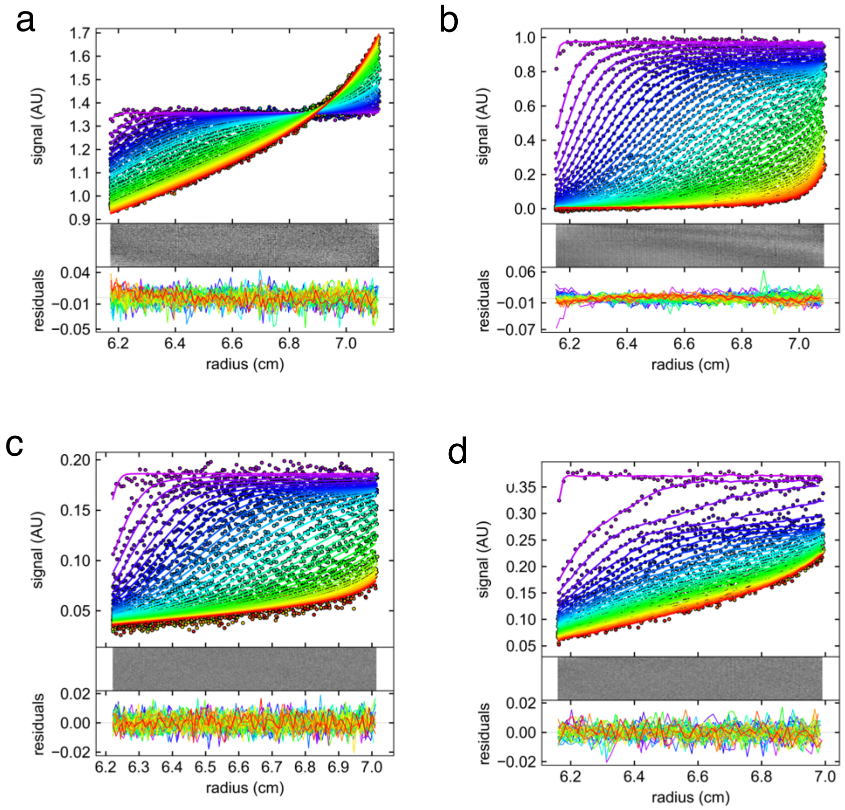

Figure S4: SV-AUC runs showing raw data (points) and fits using the continuous distribution (*c(s)*) method by the program SEDFIT (solid lines) [1]. For clarity, every third dataset is shown. The middle residual bitmap plot and the lower panel show residuals derived from the fit (a) 300 μM EGCG, (b) 30 μM P53-NTD, (c)

30 µM EGCG + 30 µM p53-NTD, (d) 30 µM p53-NTD + 300 µM EGCG. All samples were collected in 3 mm pathlength centerpieces with the exception of B where the centerpiece pathlength was 12 mm.

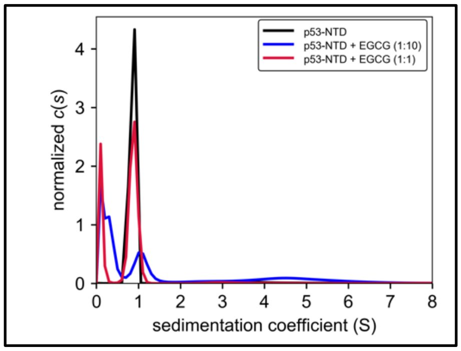

Figure S5. Diffusion-deconvolved sedimentation coefficient distributions (*c(s)*) for p53-NTD at the indicated ratio of EGCG:NTD, showing EGCG-dependent shift and broadening of the NTD peak.

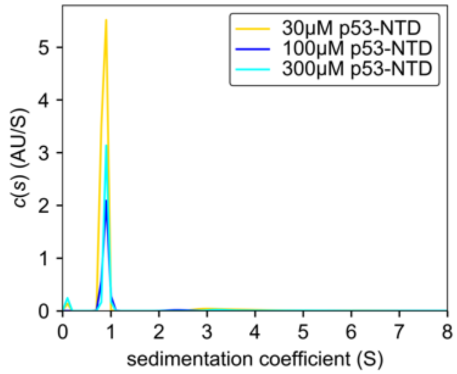

Figure S6. Diffusion-deconvolved sedimentation coefficient distributions (*c(s)*) for p53-NTD at the indicated concentrations, showing monodispersity of NTD monomer.

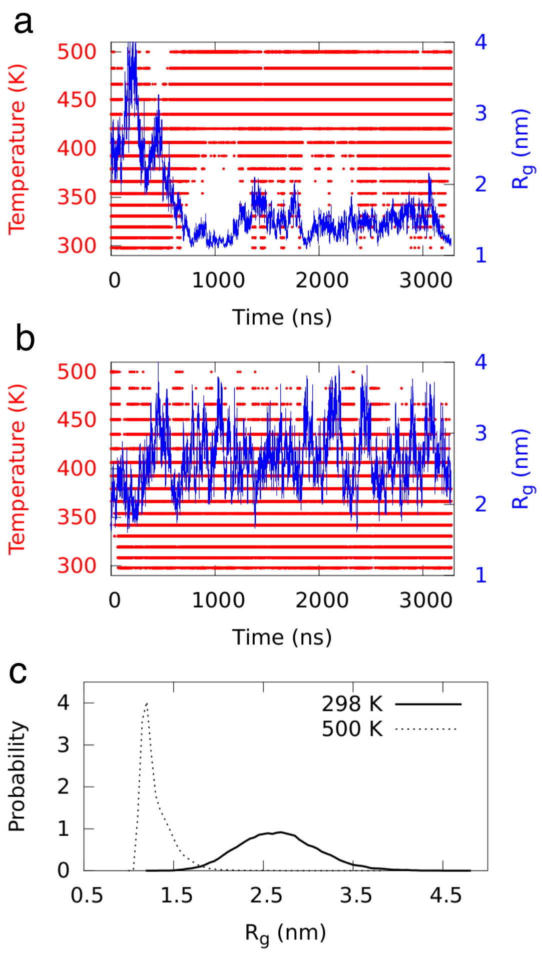

Figure S7. (a and b) Effective solute temperature (red traces) and Rg of p53-NTD (blue traces) as a function of simulation time for two representative replicas in NTD REST2 simulations. (c) Probability distribution of NTD R_g under two different conditions in REST2 simulation: effective solute temperature of 298 K for unscaled Hamiltonian and 500 K for scaled Hamiltonian of λ=0.6.

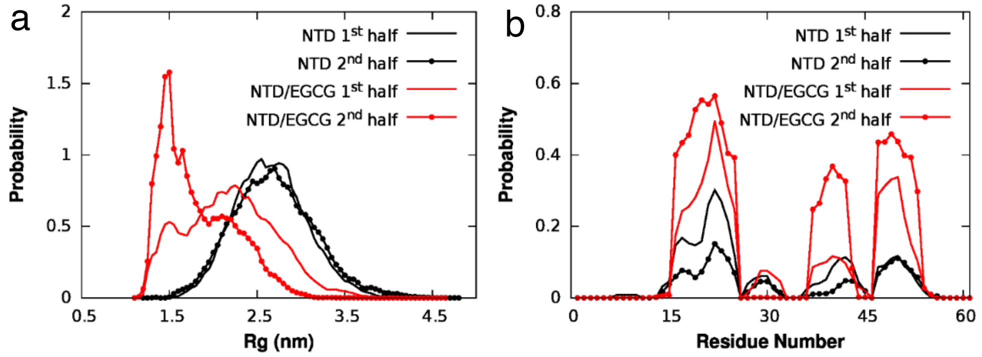

Figure S8. Convergence of NTD and NTD/EGCG simulations at the level of R_g (a) and α-helical probability (b). Each production simulation trajectory was divided in the middle into two equal halves before calculation.

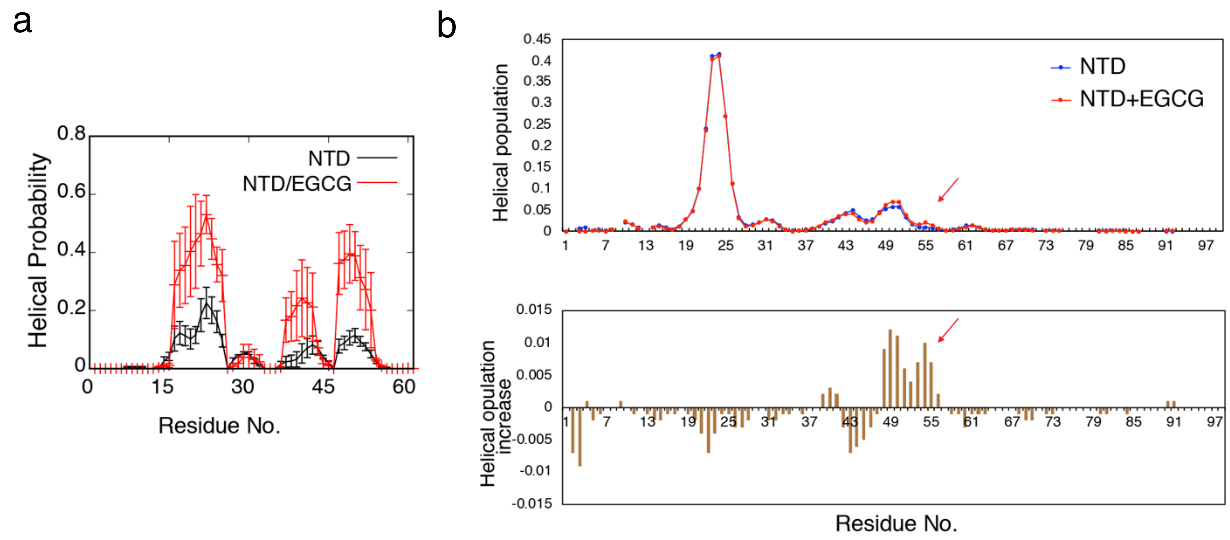

Figure S9. EGCG increases helical propensities of NTD. (a) α-helical probability of p53-NTD residues in the presence or absence of EGCG calculated from REST2 simulations. Uncertainties were estimated by dividing each trajectory into three segments and calculating the standard deviation of mean values, which were represented by error bars (n=3). (b) Residue based helical population increase of NTD upon EGCG binding estimated by δ2D method[2].

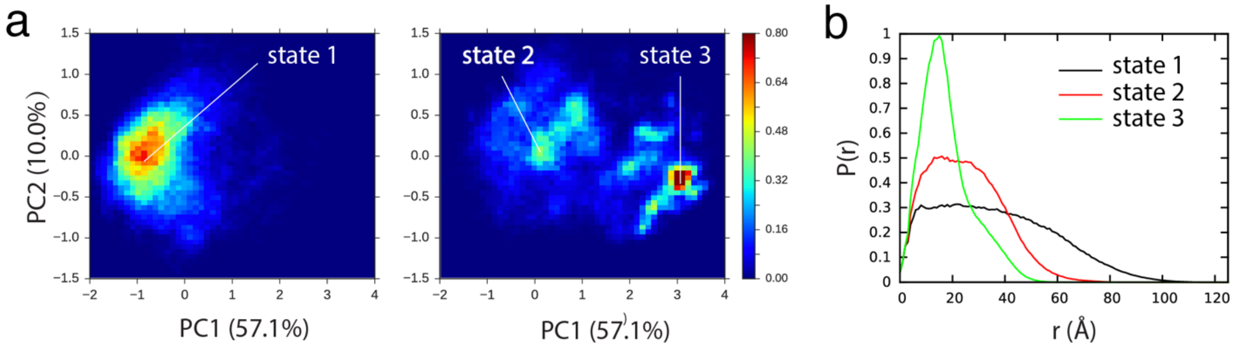

Figure S10. Pairwise distance P(r) calculated from thress highly populated NTD conformations.

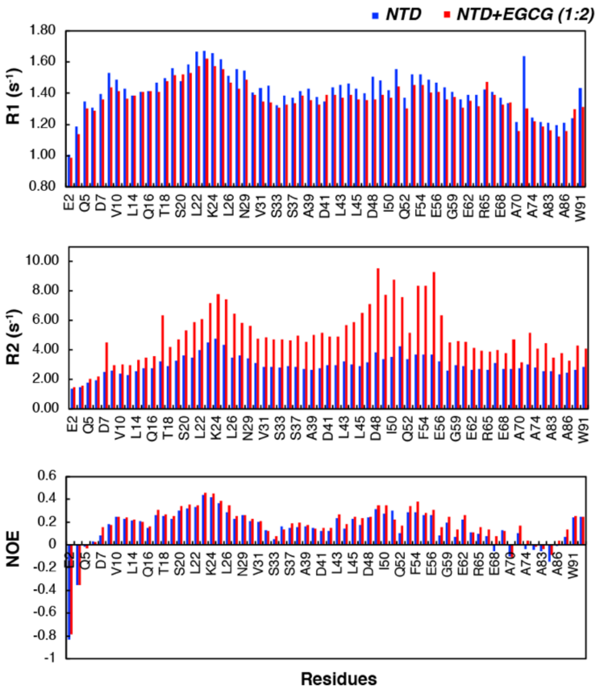

Figure S11. ^{15}N R1, R2 and ^{1}H-^{15}N heteronuclear NOE of NTD in the absence (blue) and presence (red) of EGCG.

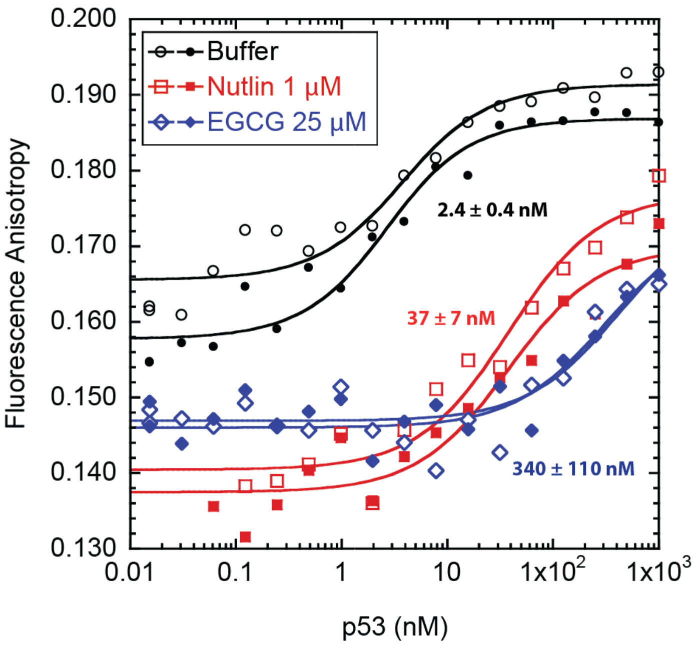

Figure S12. EGCG inhibits p53-MDM2 binding under equilibrium conditions. Fitting the fluorescence anisotropy of fluorescein-labeled MDM2 in the presence of p53 indicates a dissociation constant (KD) for the full-length proteins of 2.4 ± 0.4 nM. The addition of 25 µM EGCG inhibits this interaction, weakening the apparent KD to 340 ± 110 nM. 1 µM Nutlin 3a is included as a positive control, and weakens the observed KD to 37 ± 7 nM.

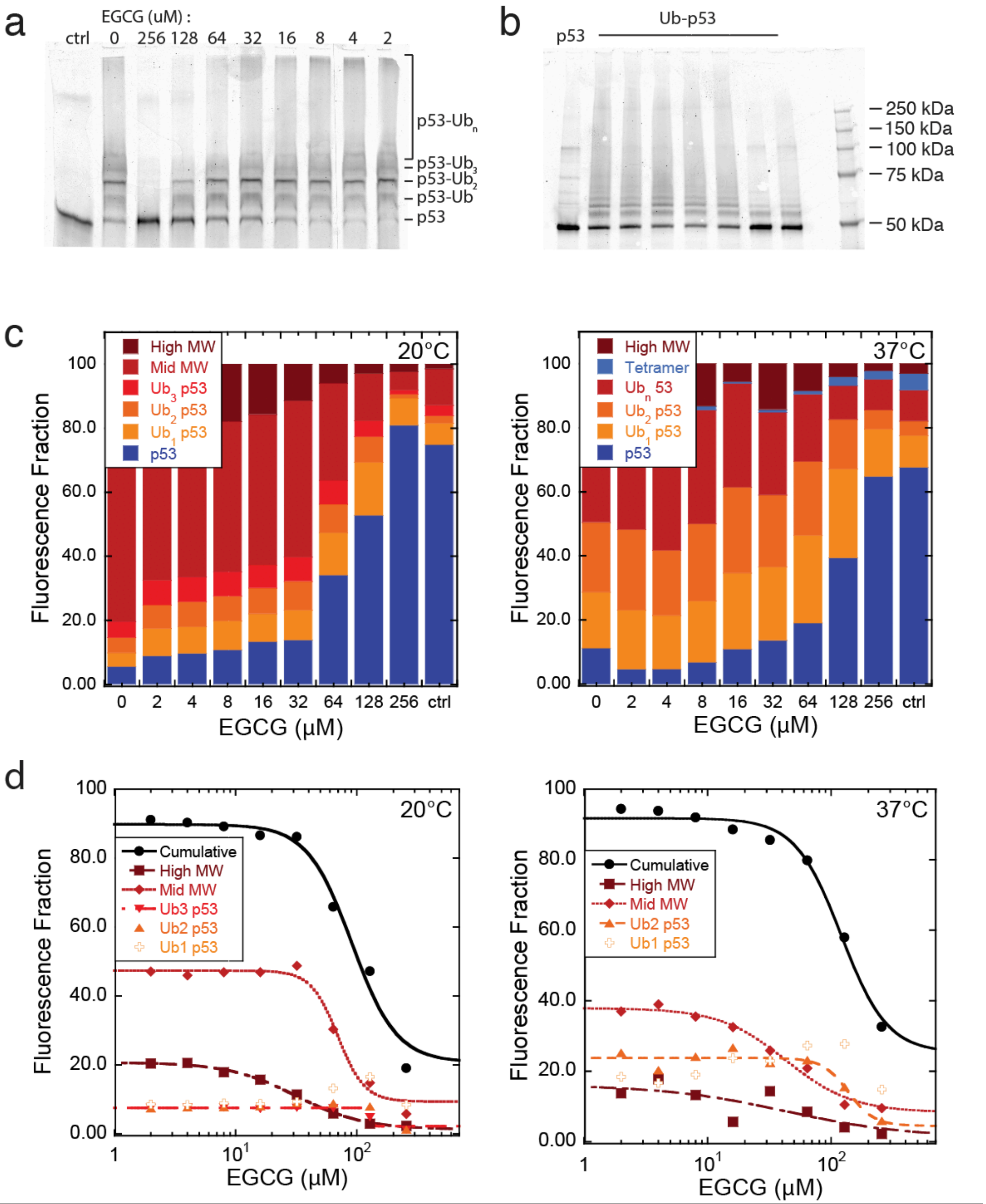

Figure S13. EGCG inhibits the ubiquitination of p53 by MDM2 *in vitro*. (a) *In vitro* ubiquitination of fluorescein-tagged p53 was carried out in the presence of up to 0.25 mM EGCG at 37 °C. The products were separated by gradient SDS-PAGE and visualized by fluorescence. (b) Ubiquitinated fluor-p53 with molecular weight marker. Sample lane 1: non-ubiquitinated p53. Lanes 2-6: ubiquitnated p53. Lanes 7,8: ubiquitinated p53, EGCG inhibited (100uM). Lane 9: Bio-Rad Precision Plus prestained marker. (c) Fluorescence densitometry was used to quantify the extent of ubiquitination in A. (d) Plotting the fluorescence fraction of modified p53 products as a function of EGCG concentration and fitting to a four-parameter sigmoids yield EGCG IC_{50} values ranging from 40 to 100 μM. The population of mono-ubiquitinated p53 is not described by a sigmoid.

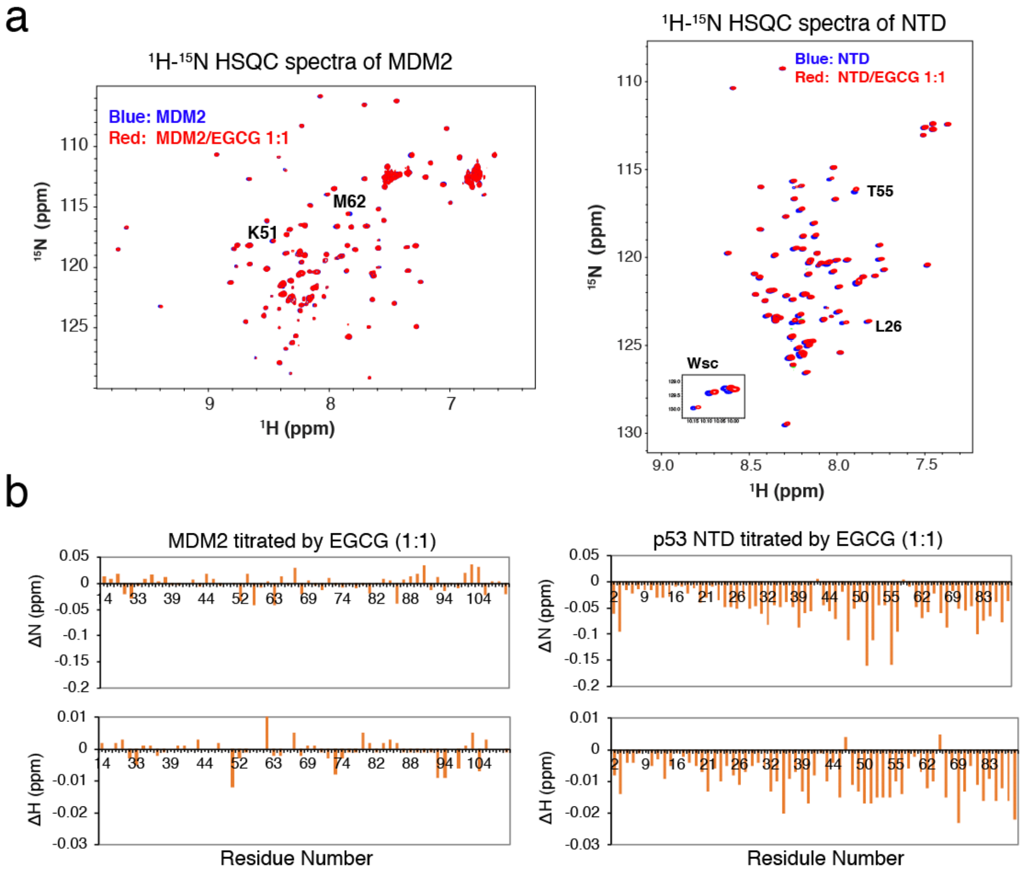

Figure S14. CSPs comparison indicated that EGCG binds to MDM2 much weaker than binds to NTD. (a). ^{1}H-^{15}N HSQC spectra of MDM2 (left) and NTD (right) perturbed by 1:1 ratio of EGCG. (b) Chemical shifts perturbations of MDM2 (left) and NTD (right) in amide nitrogen (ΔN) and amide proton (ΔH) dimension induced by EGCG.

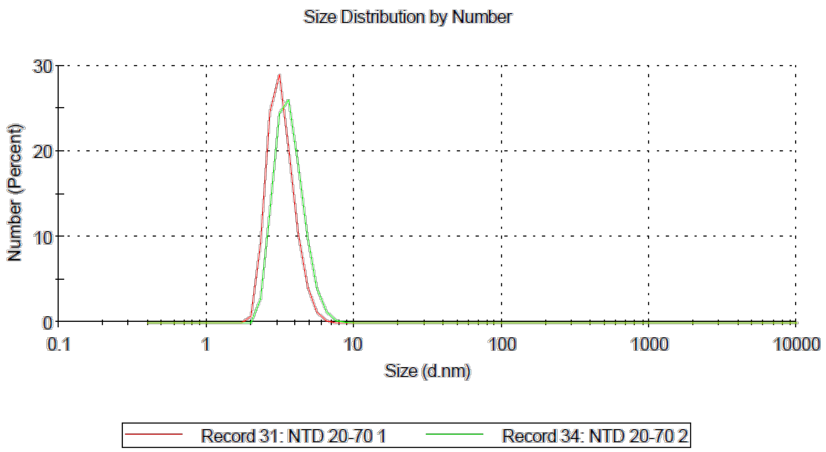

Figure S15. Dynamic light scattering (DLS) of NTD sample used in SAXS. The monodispersity was confirmed by a single peak in DLS. The red and green lines are consecutive measurements of n=12 scans each for 10 sec/scan.

Table S1. Summary of SAXS data

**Sample**	**R_G (Å)** Guinier plot	**R_G (Å)** p(r) function	**I(0)** p(r) function	**TE / χ^2** p(r) function	**D_{Max}** p(r) functio n	**MW (kDa)**
NTD (580 μM)	23.9 ±0.5	25.5 ± 0.5	0.0298 ±0.0002	0.61/1.07	122	6.4
NTD/EGCG 1:1	24.0 ±0.4	24.9 ± 0.5	0.0310 ±0.0001	0.62/1.06	123	6.5
NTD/EGCG 1:2	23.9 ±0.4	26.5 ± 0.6	0.0333 ±0.0001	0.60/1.07	129	6.5

R_G: radius of gyration. I(0): scattering intensity at zero angle. TE: total estimate. D_{Max}: maximum particle diameter. MW: molar mass. Molar masses were determined with the Size & Shape method[3]. The calculated MW for a NTD monomer is 6.9 kDa.

References

1. Schuck, P. Size-distribution analysis of macromolecules by sedimentation velocity ultracentrifugation and Lamm equation modeling. *Biophys. J.* **78**, 1606–1619 (2000).
2. Camilloni, C., De Simone, A., Vranken, W. F. & Vendruscolo, M. Determination of secondary structure populations in disordered states of proteins using nuclear magnetic resonance chemical shifts. *Biochemistry* **51**, 2224–2231 (2012).
3. Hajizadeh, N. R., Franke, D., Jeffries, C. M. & Svergun, D. I. Consensus Bayesian assessment of protein molecular mass from solution X-ray scattering data. *Sci. Rep.* **8**, (2018).
