# Supplementary material for: EGCG binds intrinsically disordered N-terminal domain of p53 and disrupts p53-MDM2 interaction
Source: Nat Commun. 2021 Feb 12;12:986. doi: 10.1038/s41467-021-21258-5 (PMC7881117; doi:10.1038/s41467-021-21258-5)
Supplement: Supplementary file 3 — Reporting Summary [file 41467_2021_21258_MOESM3_ESM.pdf]

nature research

Corresponding author(s): Chunyu Wang
Last updated by author(s): Nov 17, 2020

Reporting Summary

Nature Research wishes to improve the reproducibility of the work that we publish. This form provides structure for consistency and transparency in reporting. For further information on Nature Research policies, see our Editorial Policies and the Editorial Policy Checklist.

Statistics

For all statistical analyses, confirm that the following items are present in the figure legend, table legend, main text, or Methods section.

n/a	Confirmed	
☐	☒	The exact sample size (*n*) for each experimental group/condition, given as a discrete number and unit of measurement
☒	☐	A statement on whether measurements were taken from distinct samples or whether the same sample was measured repeatedly
☒	☐	The statistical test(s) used AND whether they are one- or two-sided<br>*Only common tests should be described solely by name; describe more complex techniques in the Methods section.*
☒	☐	A description of all covariates tested
☒	☐	A description of any assumptions or corrections, such as tests of normality and adjustment for multiple comparisons
☐	☒	A full description of the statistical parameters including central tendency (e.g. means) or other basic estimates (e.g. regression coefficient) AND variation (e.g. standard deviation) or associated estimates of uncertainty (e.g. confidence intervals)
☒	☐	For null hypothesis testing, the test statistic (e.g. *F*, *t*, *r*) with confidence intervals, effect sizes, degrees of freedom and *P* value noted<br>*Give P values as exact values whenever suitable.*
☒	☐	For Bayesian analysis, information on the choice of priors and Markov chain Monte Carlo settings
☒	☐	For hierarchical and complex designs, identification of the appropriate level for tests and full reporting of outcomes
☒	☐	Estimates of effect sizes (e.g. Cohen's *d*, Pearson's *r*), indicating how they were calculated

Our web collection on statistics for biologists contains articles on many of the points above.

Software and code

Policy information about availability of computer code

Data collection	Biacore 3000 control software, topspin 3.5pl7, GROMACS 2019, DYNAMICS 7.1
Data analysis	Topspin 4.0.6, Sparky 3.115, BIAevaluation 4.0.1, GROMACS 2019, BioXTAS RAW (1.6.3 and 1.6.4), UCSF Chimera 1.9, BioRad ImageLab 6.1, ATSAS 3.0.0-3 software suite

For manuscripts utilizing custom algorithms or software that are central to the research but not yet described in published literature, software must be made available to editors and reviewers. We strongly encourage code deposition in a community repository (e.g. GitHub). See the Nature Research guidelines for submitting code & software for further information.

Data

Policy information about availability of data

All manuscripts must include a data availability statement. This statement should provide the following information, where applicable:
- Accession codes, unique identifiers, or web links for publicly available datasets
- A list of figures that have associated raw data
- A description of any restrictions on data availability

The data that support the findings of this study are available from the corresponding author upon reasonable request.

Field-specific reporting

Life sciences study design

All studies must disclose on these points even when the disclosure is negative.

Sample size	No statistical method was used to predetermine sample size. Sample size was chosen to ensure reproducibility and to estimate errors in the measurement.
Data exclusions	No data was excluded in the analysis
Replication	For SPR analysis, all samples were flowed through 3 different channels. Replications efforts were successful. For NMR analysis, resonance peaks in the spectra are reproduced well.
Randomization	Not applicable. Randomization used in clinical trials and animal studies is usually not necessary in biophysical studies, where the measurement difference from control is very significant and there are little placebo effects.
Blinding	The investigators were not blinded to allocation during experiments. Blinding used in clinical trials and animal studies is usually not necessary in biophysical studies, where the measurement difference to control is very significant and there are little placebo effects.

Reporting for specific materials, systems and methods

We require information from authors about some types of materials, experimental systems and methods used in many studies. Here, indicate whether each material, system or method listed is relevant to your study. If you are not sure if a list item applies to your research, read the appropriate section before selecting a response.

Materials & experimental systems

n/a	Involved in the study	
☒	☐	Antibodies
☒	☐	Eukaryotic cell lines
☒	☐	Palaeontology and archaeology
☒	☐	Animals and other organisms
☒	☐	Human research participants
☒	☐	Clinical data
☒	☐	Dual use research of concern

Methods

n/a	Involved in the study	
☒	☐	ChIP-seq
☒	☐	Flow cytometry
☒	☐	MRI-based neuroimaging
